# Sero-epidemiological evaluation of changes in *Plasmodium falciparum *and *Plasmodium vivax *transmission patterns over the rainy season in Cambodia

**DOI:** 10.1186/1475-2875-11-86

**Published:** 2012-03-25

**Authors:** Jackie Cook, Nico Speybroeck, Tho Sochanta, Heng Somony, Mao Sokny, Filip Claes, Kristel Lemmens, Michael Theisen, Irene S Soares, Umberto D'Alessandro, Marc Coosemans, Annette Erhart

**Affiliations:** 1 Institute of Tropical Medicine, Nationalestraat 155, Antwerp 2000, Belgium; 2 Ecole de santé publique, Université Catholique de Louvain, Clos Chapelle-aux-Champs, Brussels 1200, Belgium; 3 National Center for Malaria Control, Parasitology and Entomology, Phnom Penh, Cambodia; 4 Centre for Medical Parasitology at Department of International Health, Immunology and Microbiology, University of Copenhagen, Copenhagen, Denmark; 5 Departamento de Análises Clínicas e Toxicológicas, Faculdade de Ciências Farmacêuticas, Universidade de São Paulo, São Paulo State, Brazil; 6 Disease Control and Elimination, Medical Research Council Unit, Fajara, The Gambia

**Keywords:** Malaria, Serology, Classification and regression tree, Elimination, Cambodia

## Abstract

**Background:**

In Cambodia, malaria transmission is low and most cases occur in forested areas. Sero-epidemiological techniques can be used to identify both areas of ongoing transmission and high-risk groups to be targeted by control interventions. This study utilizes repeated cross-sectional data to assess the risk of being malaria sero-positive at two consecutive time points during the rainy season and investigates who is most likely to sero-convert over the transmission season.

**Methods:**

In 2005, two cross-sectional surveys, one in the middle and the other at the end of the malaria transmission season, were carried out in two ecologically distinct regions in Cambodia. Parasitological and serological data were collected in four districts. Antibodies to *Plasmodium falciparum *Glutamate Rich Protein (GLURP) and *Plasmodium vivax *Merozoite Surface Protein-1_19 _(MSP-1_19_) were detected using Enzyme Linked Immunosorbent Assay (ELISA). The force of infection was estimated using a simple catalytic model fitted using maximum likelihood methods. Risks for sero-converting during the rainy season were analysed using the Classification and Regression Tree (CART) method.

**Results:**

A total of 804 individuals participating in both surveys were analysed. The overall parasite prevalence was low (4.6% and 2.0% for *P. falciparum *and 7.9% and 6.0% for *P. vivax *in August and November respectively). *P. falciparum *force of infection was higher in the eastern region and increased between August and November, whilst *P. vivax *force of infection was higher in the western region and remained similar in both surveys. In the western region, malaria transmission changed very little across the season (for both species). CART analysis for *P. falciparum *in the east highlighted age, ethnicity, village of residence and forest work as important predictors for malaria exposure during the rainy season. Adults were more likely to increase their antibody responses to *P. falciparum *during the transmission season than children, whilst members of the Charay ethnic group demonstrated the largest increases.

**Discussion:**

In areas of low transmission intensity, such as in Cambodia, the analysis of longitudinal serological data enables a sensitive evaluation of transmission dynamics. Consecutive serological surveys allow an insight into spatio-temporal patterns of malaria transmission. The use of CART enabled multiple interactions to be accounted for simultaneously and permitted risk factors for exposure to be clearly identified.

## Background

Malaria transmission is often focal, particularly in low endemic areas. Entomological and parasitological measures are traditionally used to estimate its intensity, though in areas of low transmission the required sample sizes for entomological surveys increase exponentially because of the difficulty of finding infected mosquitoes. In addition, parasitological surveys using microscopy can be extremely time consuming and may not detect sub-patent infection [1]. Serological indices have demonstrated their use as an informative additional measure [2] and population level sero-prevalence to specific malarial antigens can be used to estimate the force of infection - the rate at which individuals become infected - in a given area [3]. These techniques have also been exploited to document changes in transmission intensity [4], to identify 'hotspots' of malaria transmission [5,6] and, historically, to confirm elimination in Greece and Mauritius [7,8].

Where malaria transmission is seasonal, the characterization of its dynamics requires the longitudinal collection of the variables of interest (entomological or parasitological). As antibodies remain in the blood longer than parasites, they are less subject to seasonal variations. Whilst individual responses may fluctuate [9-12], previous studies suggest that population sero-prevalence remains similar if transmission intensity remains consistent over the years [13].

Cambodian malaria control programmes have historically focussed on the border area between Thailand and Cambodia, which was one of the sites of emergence of chloroquine and, more recently, of artemisinin resistance, increasing the urgency for containment and/or elimination. One of the long-term challenges Cambodia faces is forest-related malaria and the residual transmission amongst high-risk populations (ethnic minorities, migrant workers) in remote areas [14,15]. Malaria transmission within Cambodia, as across the whole Mekong region [16], is highly heterogeneous [15] and the presence of both *Plasmodium falciparum *and *Plasmodium vivax *malaria compounds the difficulties of estimating transmission intensity and controlling the disease [17-20].

**Figure 1:**
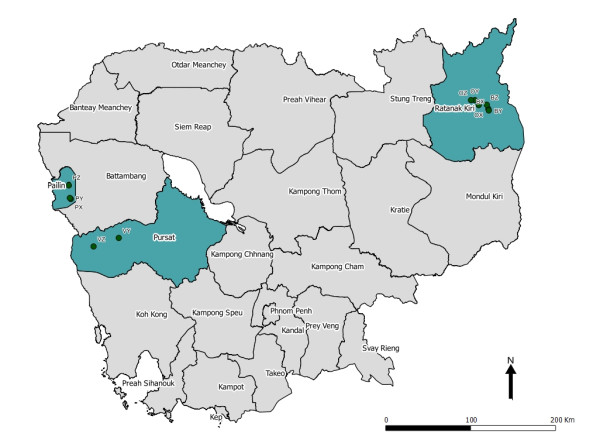
**Map of study sites**.

The CAMALFOR project was a joint collaboration between the Institute of Tropical Medicine in Antwerp, Belgium and the National Center for Malaria Control, Parasitology and Entomology (CNM) in Phnom Penh, Cambodia. In 2005, two surveys, one in the middle and the other at the end of the transmission season, were carried out in four districts: Borkeo and O'Chum in the east and Pailin and Veal Vang in the west (Figure 1). Entomological, parasitological and serological data were collected to characterize the malaria transmission over time in these areas. This paper reviews the use of serological measures for estimating transmission intensity and identifying risk factors for malaria sero-conversion using Classification and Regression Trees (CART).

## Methods

### Study population and site

Two malariometric cross-sectional surveys were performed in four districts of Cambodia: two in the eastern region (Borkeo and O'Chum in Rattanakiri Province) and two in the western region (Veal Vang in Pursat Province and Mittapheap in Pailin Province), in August and November 2005 (Figure 1). In each district, the study was conducted in three forest villages with different degrees of deforestation, except in Pailin where only cleared forest remained. Ethnic minority groups (Charay and Tumpurn) were present in both eastern districts, whilst the western region was inhabited primarily by Khmer. In both regions, the dry season typically runs from November to May and the rainy season from June to October. Both *P. falciparum *and *P. vivax *malaria were present and *Anopheles dirus sensu stricto *was found to be the major malaria vector in these study villages, with *Anopheles minimus s.s *and *Anopheles barbirostris *representing minor vectors [21].

The study was originally designed to evaluate malaria transmission intensity in order to plan targeted interventions. In each of the four "forested" districts, three villages (represented by X, Y and Z) were selected for their representativeness of forest coverage in the area. Each village consisted of approximately 200 inhabitants, of which 100 were randomly sampled for the survey. However, in Veal Vang district (Pursat Province, western region) bloodspot filter papers from one of the villages (VX) were stored incorrectly and therefore could not be analysed for the serological survey. Hence, this village was not included in the analysis. For direct comparison of antibody responses and associated risks across the second part of the rainy season (August to November), only individuals who took part in both surveys were analysed in this paper. Analyses for each of the two regions have been kept separate throughout the paper.

### Data collection and management

#### Census and survey

A full census of the study population collecting information on age, gender, ethnicity, education, housing conditions, socio-economic level, forest-related activities and malaria prevention measures was carried out in July 2005. The cross -sectional surveys (August and November) included a random sample of 100 individuals per village - this sample size was chosen to provide a precision of 3% for a minimum estimated parasite index of 5%. Each participant had thick and thin slides taken for microscopic examination, as well as having a filter paper blood spot collected for malaria antibody detection. Everyone was examined for clinical signs of malaria and a rapid diagnostic test (RDT) was administered to participants with symptoms. Malaria treatment was based on the RDT results and followed the National guidelines. A new variable combining ownership of bicycle, TV and motorbike was generated using principal component analysis in order to estimate each family's income level (highest, medium, low, lowest), as described previously [22]. Bed net usage was classified as sufficient if there was at least one bed net for two people in each household.

### Laboratory procedures

#### Parasitological measures

Parasite prevalence was determined by microscopy. Slides were stained for 15 minutes with a 10% Giemsa solution. Thick films and thin films were taken from each participant and parasite density was computed based on the examination of 200 white blood cells and assuming a white blood cell count of 8,000/μl. Microscopy was performed at the National Center for Entomology, Parasitology and Malariology (CNM) in Phnom Penh, Cambodia, and quality control was done on 10% of randomly chosen slides by a second reader. Any discordant slides were re-read by both readers until an agreement was found.

### Serological measures

A blood spot was taken on filter paper (Whatman grade 3) and allowed to dry before being individually stored in a zipped plastic bag containing silica gel and kept at -20°C until ELISA assays were performed at ITM, Antwerp. *P. falciparum *GLURP antibodies and *P. vivax *MSP-1_19 _antibodies were detected using ELISA. Briefly, from each blood spot, a disc of 5 mm diameter was punched out and eluted overnight at 4°C in 2 ml of PBS-Blotto-Tween (0.01 M phosphate, 0.2 M NaCl, 0.05% w/v NaN_3_, 1% w/v skimmed milk powder, 0.05% v/v Tween 20, pH 7.4). Two hundred μl of the eluate was added in duplicate to blocked ELISA plates coated separately with *P. falciparum *GLURP R2 [23] and *P. vivax *MSP-1_19 _[24]. Pooled sera from five *P. falciparum *or *P. vivax *infected patients and from five non-infected control group were diluted at 1:400 in PBS-Blotto to serve as positive and negative control respectively. Goat anti-human IgG(H + L) peroxidase (Sigma, affinity purified) was diluted to 1:20,000 in PBS-Tween and incubated for 1 h before development of the ELISA using 200 μl ABTS substrate-chromogen solution. Optical Densities (ODs) were read at 415 nm (Elx808, BioTek Instruments). Corrected OD values were obtained by subtracting the mean OD of the antigen negative control wells from the mean OD of the corresponding antigen containing wells. Subsequently, the percent positivity (PP) of each specimen was calculated using the positive control serum' OD as 100%. This ensured the sample results were standardized across ELISA plates. A mixture model was used to generate a cut off for positivity as previously described [2].

### Statistical methods

All data were double entered, validated and cleaned in Epi Info 6 (CDC, USA). The dataset was analysed using STATA v.11 (Statacorp, Texas, USA) and CART (Salford Systems, USA) software.

### Force of infection

A simple reversible catalytic conversion model was used to fit the dichotomized serological results, using maximum likelihood methods [3,13]. The model produces a force of infection (FOI) or sero-conversion rate (SCR or λ) and a sero-reversion rate (ρ). For this paper, the sero-conversion rates are presented with the sero-prevalence curves and the sero-reversion rate are presented in the text. Profile likelihood plots, as previously described, were examined to detect any indication of increased force of infection (λ) at a particular age within the population [4]. Sero-prevalence curves were fitted with two forces of infection if deemed necessary by likelihood ratio test (p < 0.05).

### Changes in antibody responses over season

To evaluate changes in antibody responses during the rainy season, analysis focussed on *P. falciparum *results from the eastern surveys as this area experienced the biggest serological differences between the surveys. Wilcox paired rank tests were used to test for differences in antibody responses between August and November (p < 0.05 considered significant).

The use of Classification and Regression Trees (CART) is described extensively elsewhere [22,25]. This method was utilized to overcome some of the difficulties of dealing with multiple interactions and collinearity in multi-variate models. The models used in CART are non-linear and non-parametric. The software successively splits the dataset into homogeneous subsets based on the target of interest, in this case malaria sero-positivity. A regression model was used in CART which allowed for the identification of 'clusters' of homogenous responses in the magnitude of the antibody response for each survey; these populations were subsequently classified into four sero-positivity categories: i) negative, ii) low positive, iii) positive, and iv) highly positive. Individual changes in sero-positivity categories, between the August and November surveys, were analysed using a categorical model in CART and classified as follows: i) remaining negative or decreasing one category (0 or -), ii) people remaining in the same sero-positive category (1 or +), iii) people increasing one category (2 or ++) and, iv) people increasing two or three categories (3 or +++). Predictor variables included in the models were age (continuous), sex, ethnic group, profession, owning a forest plot, owning a forest plot house, wall and roof type of house, education, bed net usage, income level and village. A tree within one standard error of minimal cost was accepted. As well as generating decision trees, CART also produces a table indicating the overall importance of each of the predictors entered into the model, reporting a discriminatory power ranging from 0 to 100%. In some cases, the predictors in the table will not appear in the tree as only the best predictor which creates the most homogenous sub-groups at each node is displayed. However, the overall importance of each variable is assessed on its discriminatory power for the total number of nodes in the tree and reported in the text.

### Ethical clearance

Permission was received from village leaders after explaining the study aims and objectives. Oral informed consent was obtained from all adults and parents/guardians. Ethical clearance was obtained from the CNM, Phnom Penh and the IRB of the ITM, Antwerp (n° OG018). Permission was also obtained from the Cambodian Ministry of Health.

## Results

Individuals who participated in both surveys (N = 804) constituted approximately 50% of the population originally surveyed (547 only surveyed in August, 213 only surveyed in November). No bias in terms of census information was detected in this sample, although the Pore ethnic group in the western region (originally representing less than 2.5% of the population according to the census) did not participate in both surveys, so were not included in this analysis. Baseline data of individuals who participated in both surveys is summarized in Table 1. Among the 804 participants analysed, 384 lived in the eastern region and 420 lived in the western region. The population was young (median age of 17 and 18 years in the east and west, respectively) and mainly consisted of the Charay and Tumpurn ethnic groups in the east whilst the western surveys included solely Khmer individuals. Education level was low especially in the east where most of the adult population (84%) was illiterate, whilst in the west, almost half (44%) had received primary education. Housing structure also differed between the two regions with the majority being made from bamboo or leaf (98%) in the east, whilst house material was much more varied in the west. Bed net use was particularly low in the east where only 27% of the respondents reported living in a house with sufficient net coverage, whilst it was much higher (62%) in the west. The majority of participants reported being part of a family who owned a forest plot (92% - 353/384 in the east; 86% - 363/420 in the west), with fewer people reporting owning a forest plot house (74% - 285/348 in the east; 57% - 239/420 in the west). Additionally, the majority of participants over the age of 15 reported working in the forest (89% -174/195 in the east; 90% - 207/229 in the west).

**Table 1:** Baseline characteristics of the study population (N = 804)

	East (N = 384)		West (N = 420)	
	**n**	**%**	**n**	**%**

**Sex**				

Female	207	53.9	235	56

**Ethnicity**				

Khmer	30	7.8	420	100

Charay	175	45.6		

Tumpurn	179	46.6		

Education*				

None	323	84.1	183	43.6

Primary	58	15.1	186	44.3

Secondary	1	0.3	50	11.9

**Do you use a bed net?**				

No	101	26.3	3	0.7

Yes, sufficient	103	26.8	261	62.1

Yes, not sufficient	180	46.9	156	37.1

**Income quartile**				

Lowest	184	43.8	208	54.2

Low	48	11.4	63	16.4

Medium	82	19.5	36	9.4

Highest	106	25.2	77	20.1

**Wall type of house**				

Thatch	4	1.0	61	14.5

Bamboo	234	60.9	82	19.5

Leave	141	36.7	175	41.7

Iron	0	0	22	5.2

Dried Mud	0	0	19	4.5

Other e.g wood	5	1.3	61	14.5

**Roof type of house**				

Thatch	212	55.2	272	64.8

Bamboo	25	6.5	17	4.1

Leave	12	3.1	29	6.9

Iron	133	34.6	99	23.6

Dried mud	2	0.5	3	0.7

Do you work in the forest?** (> = 15 yrs old)				

Yes	207/229	90.0	174/195	89.0

**Do you own a forest plot?**				

Yes	353	91.9	363	86.4

**Do you own a forest plot house?**				

Yes	285	74.2	239	56.9

* 2 missing in August, 1 missing in November

** 1 missing in August, 1 missing in November

Malariometric indices indicated clear differences in malaria transmission patterns between the east and west regions (Table 2). In the east, *P. falciparum *was twice as prevalent as *P. vivax*, and both parasite prevalences decreased by more than half between August and November (from 8.1% to 3.4% (p = 0.014) and from 4.4% to 2.1% (p = 0.077), for *P. falciparum *and *P. vivax *respectively). During this same period, *P. falciparum *sero-prevalence increased more than two-fold (from 23% to 49% (p < 0.001)) while *P. vivax *sero-prevalence remained similar (12.8% and 11.2% in August and November respectively (N.S)). In the western region, *P. vivax *parasite prevalence was almost 10-fold higher than *P. falciparum *(10.1% *versus *1.2%) and did not decrease significantly between August and November (from 10.7% to 9.1% (N.S)) whereas *P. falciparum *prevalence decreased by more than half to reach 0.5% (N.S) in November. *P. falciparum *sero-prevalence also decreased by half, from 14% to 7% (p < 0.001) and *P. vivax *sero-prevalence decreased from 20% to 13% (p < 0.001).

**Table 2:** Malariometric indices for the east and the west regions, in August and November 2005

	East				West			
	**August (N = 384)**		**November (N = 384)**		**August (N = 420)**		**November (N = 420)**	

	**n**	**%**	**n**	**%**	**n**	**%**	**n**	**%**

*P. falciparum *parasite*								

Positive	31	8.1	13	3.4	5	1.2	2	0.5

*P. vivax *parasite*								

Positive	17	4.4	8	2.1	45	10.7	38	9.1

***P. falciparum *serology**								

Positive	88	22.9	189	49.2	57	13.6	30	7.1

***P. vivax *serology**								

Positive	49	12.8	43	11.2	85	20.2	56	13.3

* 11 missing in August, 16 missing in November in the east; eight missing in August, 27 missing in November in the west

There was no correlation between individuals' antibody responses to the two species (p > 0.1), indicating an absence of cross-reactivity between the two antigens and suggestive of separate transmission patterns for the two species.

### Force of infection

**Figure 2:**
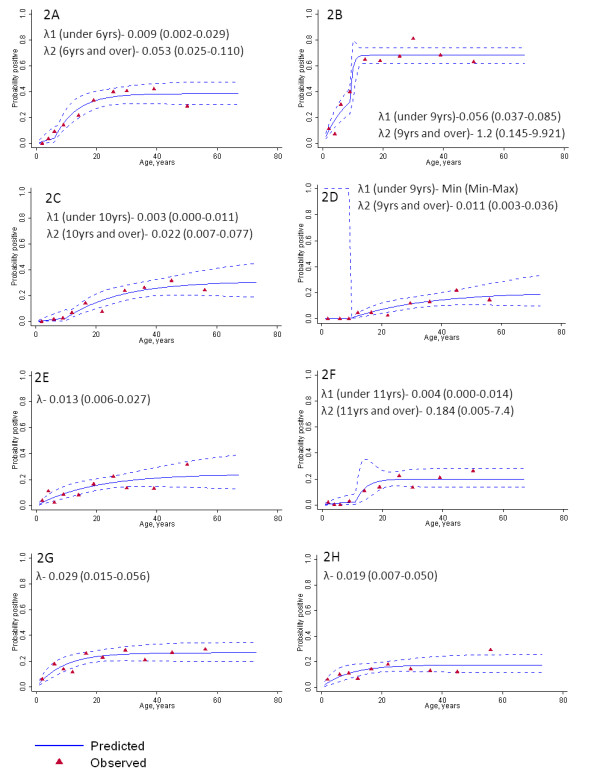
***Plasmodium falciparum *and *Plasmodium vivax *sero-prevalence curves in August and November in the eastern and western region**. Sero-prevalence curves for *P. falciparum *[2A and 2B] and *P. vivax *[2E and 2F] in the eastern region and in the western region [2C and 2D, 2G and 2H] in August and November 2005. Red triangles represent actual data points, whilst the blue lines represent the maximum likelihood model and 95% confidence intervals. Estimated force of infection (λ) is plotted on the graphs. Two forces infection, along with the age of change, are plotted if deemed necessary by likelihood ratio tests.

The age sero-prevalence curves demonstrate the different dynamics between the two species and additionally between the two regions (Figure 2, panel A to F). In the eastern region, two forces of infection (λ_1 _and λ_2_) were detected for *P. falciparum *(LR test p < 0.05) in August and November (Figure 2A and 2B), with an increase in SCR at six and nine years old for August and November respectively. The force of infection in the youngest age groups increased from 0.009 to 0.056 between August and November (six-fold increase), whilst in the older age groups it increased from 0.053 to 1.2 (20-fold increase). The sero-reversion rate was 0.084 in August and 0.053 in November, although the confidence intervals overlapped for the two surveys. The force of infection for *P. vivax *was noticeably lower than for *P. falciparum*, and there was no evidence for a higher force of infection in adults in the August survey (Figure 2E). However, in November (Figure 2F), a higher force of infection was detected in individuals over the age of 11, with a very low force of infection in children under 11 (only two sero-positives were detected, compared to nine in August). The sero-reversion rate was 0.040 and 0.051 in August and November respectively.

In the western villages, the force of infection decreased slightly between August and November for both species. For *P. falciparum*, similarly to the eastern region, two forces of infection were detected in both August and November, with increased transmission in the population over nine and 10 years old, respectively (Figure 2C and 2D). The *P. falciparum *force of infection in the youngest age groups was minimal, with only two sero-positives detected under the age of 10 in August and none in November. The sero-reversion rate was lower than in the east (0.030 and 0.028 in August and November respectively), although the confidence intervals overlapped with those in the east. A single force of infection was detected for *P. vivax *in August (Figure 2G) and by November it had decreased slightly (λ- 0.029 in August *versus *λ- 0.019 in November, Figure 2H). The sero-reversion rate was 0.080 and 0.090 in August and November respectively.

### Changes in serological response between August and November

An increase in a specific serological response is indicative of exposure to the parasite - therefore increases in antibody responses between August and November were used as a proxy for exposure. Individual responses (as measured by percent positivity (PP)) to *P. falciparum *increased significantly in the eastern region (Wilcoxon Rank Paired test, p < 0.001) between August and November, although no differences were detected for *P. vivax *responses in this area. In the west, *P. falciparum *responses did not change significantly over season; however a significant decrease was detected in *P. vivax *responses (p < 0.001).

**Figure 3:**
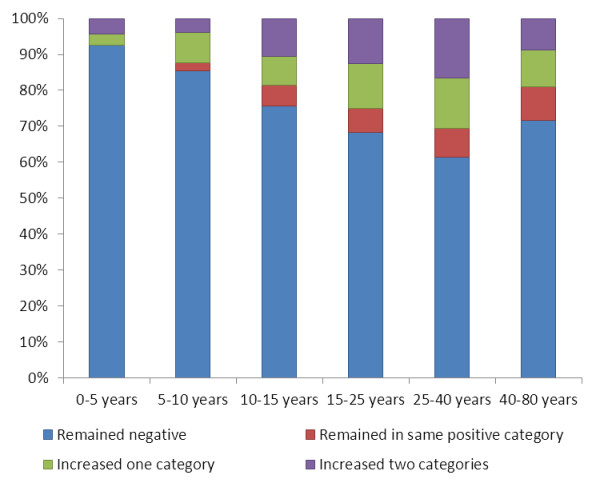
**Age breakdown of changes in *Plasmodium falciparum *serological category between August and November in the eastern region**.

In order to further investigate the changes in transmission dynamics during the rainy season, the eastern *P. falciparum *data was analysed, as the largest differences in sero-prevalence were seen in this region. CART was used to divide PP responses into four homogenous categories: negative (PP < 3.3%), low positive (PP more than 3.3% and less than 10.3%), medium positive (PP more than 10.3% and less than 24%) and high positive (PP more than 24%). Individuals were then classified depending on whether they remained negative or decreased a PP category (indicating no exposure), remained in the same positive category (indicating possible exposure) or on the number of categories they increased between August and November (indicating exposure). A total of 55% of the study population (n = 210) either remained negative or decreased a serological category between August and November whilst 8% (n = 30) remained in the same positive category, 18% (n = 70) increased one serological category and 19% (n = 74) increased two or more serological categories. Figure 3 shows the distribution of these four categories of changes according to increasing age groups. There was a clear age-dependent trend for remaining positive or increasing response between August and November, with very few changes occurring in the under five group (less than 10% sero-conversion). From the age of five onwards, there was a progressive increase in the proportion of people increasing by one category, and from the age of 10 onwards, of people increasing by two categories. Overall, there was a linear increase in the proportion of people sero-converting from less than 10% in the under-fives up to 30% in the adults aged 25 to 40, with a further 10% in this age group remaining in the same positive category.

**Figure 4:**
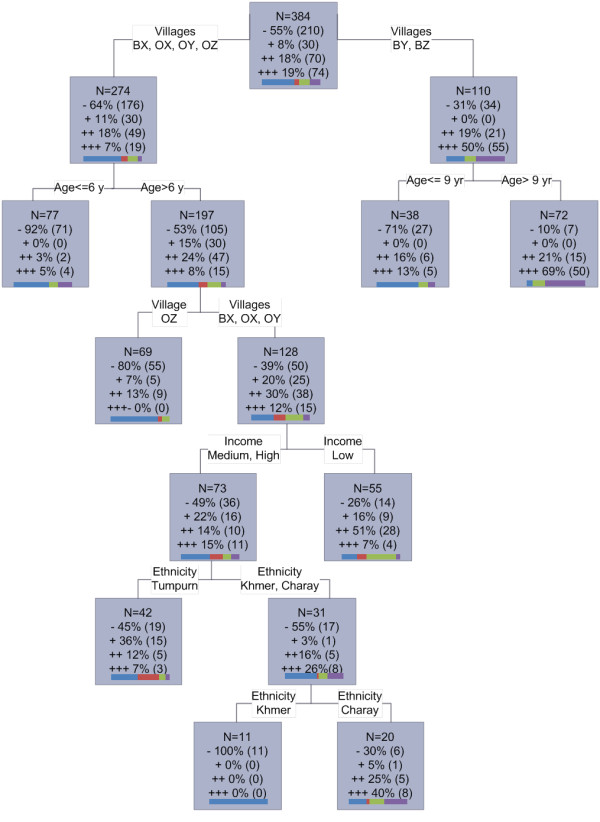
**CART analysis for risk factors associated with *Plasmodium falciparum *sero-conversion between August and November in the eastern region**. CART was performed using serological categories of increase as the outcome. - = individuals who remained negative or reduced a serological category (blue), + = individuals who remained in the same positive category (red), ++ = individuals who increased a serological category (green), +++ = individuals who increased two or more serological categories (purple). Graph bars highlight breakdown of categories in each node.

The risk factors associated with exposure to *P. falciparum *between August and November (using increase in serological category as a proxy) were analysed using CART (Figure 4). Village was the primary splitter in the tree, with BY (70% forest) and BZ (fully forested) being the most likely to increase at least one category (almost 70% of the individuals *versus *25% in the four other villages). The villages of BY and BZ were further split at the age of nine (terminal node) where 71% of under nine year olds (27/38) remained negative, compared to only 10% (7/72) of over nines. The other villages were subsequently split at age six. The under sixes remained mostly negative between the two surveys, while among the over six year olds there was another split by village with OZ (fully forested) remaining predominantly negative (80%, 55/69), and only approximately 40% (50/128) of the remaining villages (BX (deforested), OX (rubber plantation), OY (scattered forest)) remained negative. In these villages, the lowest income group was more likely to increase at least one serological category (58%) compared to medium and high income groups (29%). The latter was further split by ethnicity, first separating the Tumpurn (with 36% (15/42) remaining in a positive category), and then a further split separating the Khmer (with 100% remaining negative, n = 11) and the Charay (with 65% increasing at least one category (13/20)). The table highlighted village (100%), ethnicity (73%) and age (50%) as the three most important predictor variables, while roof type, wall type, income and working in the forest had less importance (below 20%).

## Discussion

In areas of low malaria transmission such as Cambodia, parasitological indicators of transmission intensity using microscopy can be insensitive. Serological measures allow for more sensitive detection of malaria exposure and can be used as a proxy of transmission intensity [2,4,6,26]. In this study the force of infection was estimated in different ecological settings of Cambodia and CART was used as a novel approach to analyse changes in antibody responses over time to help identify risk factors for malaria exposure during the rainy season. The results highlighted the strong heterogeneity of malaria transmission in time (throughout the rainy season) and space (at national (east/west regions) as well as local level (between villages)), suggested that adults were experiencing a higher force of infection than children and showed that CART can be a useful approach for analysing longitudinal serological data.

### Spatio-temporal heterogeneity in transmission of *Plasmodium falciparum *and *Plasmodium vivax*

Malaria parasite prevalence in the eastern and western regions was substantially different, with *P. falciparum *dominating in the east and *P. vivax *dominating in the west. This is likely to be due to the ecological differences between the two areas, such as a more intensive deforestation and lower rainfall in the western region, but also to differences in educational levels and the use of bed nets (both of which were lower in the east). *P. vivax *has been reported to become more prevalent as transmission decreases [27] and some studies have suggested that individuals with detectable *P. falciparum *infections may also have underlying *P. vivax *infections [20], which become detectable once *P. falciparum *infections are cleared through treatment. In addition, *P. falciparum *infections can sometimes mask other infections when diagnosis is performed by microscopy, meaning mixed or other malarial infections remain unrecorded. Importantly, the life cycle of *P. vivax *differs from *P. falciparum*. In *P. vivax *infections dormant liver forms (hypnozoites) can remain present for long periods of time, causing relapses months or years later. Consequently, *P. vivax *can be more difficult to eliminate and therefore may become more prominent in areas where *P. falciparum *has been successfully reduced (i.e., the western region).

CART analysis indicated that individuals in different villages in the eastern region experienced varying levels of *P. falciparum *exposure over the rainy season. Interestingly, the village OZ, characterized as a forested village, was highlighted as having little malaria exposure between August and November. It would be expected that a more forested village would be subject to higher malaria exposure. According to the entomological results obtained in the study villages, the vector densities in OZ were similar to the other villages in the eastern part of the country (Durnez *et al*, Manuscript in preparation) however, there was a particularly high usage of bed nets in this village (71% reported sufficient bed net use compared to an average of 40% in the other villages), suggesting that this may have reduced exposure successfully. Fifty percent (55/110) of the participants in villages BY and BZ (both of which are forested) increased two or more *P. falciparum *serological categories, suggesting high transmission in these villages between August and November. The serological differences between villages highlight the heterogeneity in transmission at a micro level, as has been reported previously [5].

There is also a different temporal pattern for the two species. The simple model that has been used in this study has previously been utilized in areas where sero-reversion is thought to be low [13], in which case a single survey is enough to produce an estimate of SCR for the whole year. However, in this study, substantial differences in force of infection were detected between August and November for *P. falciparum *in the eastern sites. This may be a consequence of highly seasonal yet low transmission meaning that the rarity of exposure to malaria parasites results in the loss of antibodies out of season, and any new exposure causes a large boost of antibody responses. This is demonstrated by the substantial increase in overall sero-prevalence and age sero-prevalence curves between August and November (Table 2 and Figure 2). The rates of GLURP sero-reversion (ranging between 0.05 and 0.08 in the east) predicted by the model are noticeably higher than those reported by Drakeley and colleagues [13] (where sero-reversion was fixed at 0.02), although it should be noted that different antigens are used in this study and it is likely that sero-reversion is antigen specific.

Interestingly, the parasitological measures suggested that transmission had decreased in the eastern region between August and November. This discrepancy can be explained through the delayed acquisition of antibodies and the likelihood of treatment with subsequent clearance of parasites, resulting in less parasite positive individuals by the end of the season, whilst antibody responses may still be increasing. The data is suggestive of a *P. falciparum *malaria peak, possibly occurring after August but finishing before November, resulting in lower parasitological levels, but increased sero-prevalence (Table 2). Another, not mutually exclusive, explanation for the discrepancy between the two measures is that not all current infections are detected by microscopy (sub-patent infections) [1]. A previous study in Rattanakiri in the east detected a large proportion of sub-patent infections, the majority found in adults [28]. A similarly high level of sub-patent infections was seen in a study in Central Vietnam (AE, personal communication). Sub-microscopic parasites are more likely to be present in adults who have developed partial immunity over time and may continue to stimulate production of antibodies.

In the western region *P. falciparum *SCR remained relatively steady in both surveys, although there was still evidence that adults experienced a higher force of infection. In addition, *P. vivax *force of infection did not change substantially across season in either region. A previous study in Rattanakiri also detected fluctuations in cases of *P. falciparum *throughout the year, whilst *P. vivax *cases remained steady [14]. This effect has also been reported in Vanuatu [17] and is supported by a study in Peru showing differences in seasonality between the two species, with *P. falciparum *being dominant during the wet season and *P. vivax *becoming dominant during the dry season [29]. This is likely to be a result of *P. vivax *relapses occurring in the dry season and may be an explanation for the different patterns of transmission reported in this study.

### Higher force of infection in adults

The sero-prevalence curves for *P. falciparum *consistently demonstrated a higher force of infection in adults compared to children. The age at which the increase in force of infection occurred ranged between six and 10 years old and was relatively consistent across season. For the eastern region, CART also split the data at approximately these ages with one split at nine years old and another at six years old. This suggests there was a significant difference in responses (rather than a continuous increase) between adults and children. A step in the sero-prevalence curve can be indicative of a recent reduction in transmission intensity [4]. However, in this scenario, it would be unlikely to see any large increases in antibody responses as seen in this study. Instead, the data is suggestive of adults experiencing a higher force of infection than children. In line with this, the CART analysis was able to find fewer homogenous groups in the younger age groups, suggesting the risk factors that are present for adults, are not as pertinent for children. However, in the western region, *P. falciparum *seropositivity in children was extremely low and deforestation in this area has resulted in dramatic reductions in malaria transmission. In this area, the two forces of infection may represent current transmission intensity (extremely low) and previous transmission intensity (low). Future studies in the west would allow further interpretation depending on whether the step in the seroprevalence curves remains at the same age (indicating behavioural differences) or shifts with time (indicating a drop in the force of infection).

Occupational risk of malaria in South-east Asia has already been extensively documented [14,15,22,30-32]. This study was not specifically designed to assess the effect of working in the forest on exposure and as such, it was difficult to separate the effects of age (and the increase in sero-prevalence due to cumulative exposure or biological differences) and working in the forest. Sero-positivity to *P. falciparum *in children in the western region was extremely low, suggesting they had experienced very little exposure. SCRs increased above the age of 10 years old, which is a plausible age at which children begin to accompany their parents to work in the forest. However, the sero-prevalence data indicated that SCR in children in the more forested eastern region also increased over season albeit to a lesser extent (Figure 2), suggesting transmission was not wholly attributable to occupational risk but also occurred at village level.

Another explanation for the differences in serological responses between adults and children is that immunological maturity may play a role. Children may lose their malarial specific antibodies faster than adults [12,33,34] - it is not clear if this is related to a lower cumulative exposure, i.e. having fewer episodes of antigenic stimulus, or to the immaturity of the immune system, or a combination of both). A longitudinal study in a highland area of Kenya demonstrated that children tended to lose their antibodies over the dry season, whilst adults retained high levels of antibodies throughout the year [35]. These differences between adult and child serological responses could be due to adults supporting low levels of infection through the dry season which boost immune responses, to adults having antibodies with longer half-lives, or as a result of higher levels of circulating memory cells or longer lived plasma cells [36].

CART also indicated that ethnicity played an important role in malaria exposure in the eastern region (only Khmer were analysed in the western survey). Both the Tumpurn and Charay ethnic groups appear to be at higher risk of exposure, compared to Khmer individuals. Traditionally, the ethnic minorities in this area are poorer than Khmer individuals. In the population sampled in the eastern region, 60% of Charays were classified in the poorest income group, compared with 45% of Khmer and 42% of Tumpurn. Poorer status may mean these individuals spend longer periods in the forest, and are therefore exposed for longer time periods. However, CART highlighted that ethnicity was also important in the higher income groups. This may be a reflection of the type of work undertaken by the different ethnic groups, as Khmer traditionally spend less time in the forest. Moreover in this study, the use of bed nets is much lower in the minority groups with over 50% of Charays reporting no bed nets in their household, compared with less than 1% of Khmers.

It is important to acknowledge that the serological data used in this study is based on a single antigen for each species. Although both the antigens used have been demonstrated to be relatively immunogenic [23,24,37-39], in areas of low transmission it is important to test as many antigens as possible in order to detect all possible responses. Some people do not make antibodies to particular antigens [34] and although this may not be a large problem when looking at population-based data, it could have an effect when looking at the individual level.

## Conclusion

Traditional multi-variate techniques were not suitable for the analysis of this dataset as multiple interactions and collinearity resulted in a lack of power. By using CART it was possible to investigate the data further and to clearly identify risk factors associated with malaria exposure. This was further enhanced by the analysis of consecutive serological surveys. In order to establish risk factors and to discern whether adults are experiencing a higher force of infection, only one survey would be necessary. However, to determine whether transmission is decreasing over time and eventually to determine whether elimination has occurred, more frequent surveys would be required to ensure antibody fluctuations do not result in unreliable evidence for elimination. In order to investigate antibody dynamics, samples need to be collected at more regular intervals and analyses of changes in sero-response over season, such as performed in this study, will become increasingly required.

In areas of low transmission intensity, serological indicators can be used to infer risk factors for exposure and to assess changing transmission dynamics across season and over time (and space). In areas where elimination protocols have been implemented, serological measures, in combination with molecular parasitological measures, can help to confirm areas where infection is no longer present.

## Competing interests

The authors declare that they have no competing interests.

## Authors' contributions

JC analysed the data and wrote the paper; NS contributed to the data analysis and paper review; TS supervised all the field work activities; HS contributed to the study design and fieldwork supervision; MS carried out and coordinated the fieldwork; FC supervised the sample processing with ELISA; KL processed all blood samples with ELISA; MT and IS contributed antigen to the project; UDA contributed to the study design, data analysis and reviewed the manuscript; MC contributed to the study design and coordination of all field and laboratory activities and reviewed the paper; AE contributed to the study design, the supervision of the laboratory activities, the data analysis, and reviewed the paper. All authors read and approved the final manuscript.
